# Real-Time PCR for the Diagnosis of *Acanthamoeba* Genotype T4

**DOI:** 10.3390/microorganisms10071307

**Published:** 2022-06-28

**Authors:** Aline Lamien-Meda, Martina Köhsler, Julia Walochnik

**Affiliations:** Institute for Specific Prophylaxis and Tropical Medicine, Center for Pathophysiology, Infectiology and Immunology, Medical University of Vienna, Kinderspitalgasse 15, 1090 Vienna, Austria; martina.koehsler@meduniwien.ac.at (M.K.); julia.walochnik@meduniwien.ac.at (J.W.)

**Keywords:** *Acanthamoeba*, genotype T4, qPCR, diagnostic

## Abstract

*Acanthamoeba* spp. are ubiquitous and opportunistic free-living amoebae (FLA) that can cause *Acanthamoeba* keratitis and other infections in the human host. A quick and efficient diagnosis is often challenging. Our study aimed to establish a qPCR assay to detect and, at the same time, quantify the predominant *Acanthamoeba* genotype T4. DNA from clinical corneal scrapings and *Acanthamoeba* reference strains, including genotypes T3, T4, T5, T6, T10, T11, and T12, were used to develop the new T4 assay and it was compared to published protocols and one commercial kit for evaluation. The T4 assay showed no amplification with *Acanthamoeba* genotypes T3, T5, T6, T10, T11, and T12. The efficiencies ranged from 92.01 to 97.59% (R^2^ of 0.9768 to 0.9951). The calculated LOD range was 3.63 to 33.27 cells/µL. The protocol published by Qvarnstrom and colleagues was more sensitive compared to the other assays, and an overall good agreement was observed between the new T4 and the Qvarnstrom assays. We successfully developed and validated a genotype T4 assay that could be run in duplex with the Qvarnstrom assay to reliably and simultaneously diagnose *Acanthamoeba* genotype T4 and other genotypes from clinical samples.

## 1. Introduction

*Acanthamoeba* spp. are ubiquitous and opportunistic free-living amoebae (FLA) highly abundant in soil and water, including also tap water and chlorinated swimming pools [1,2,3]. Entering the eye, they can cause a severe infection called *Acanthamoeba* keratitis (AK), with an unfavorable prognosis when mis- or late-diagnosed [4,5]. AK is a rare infection, but with a rising incidence within the past decades correlating to the increasing number of contact lens wearers [6]. *Acanthamoeba* can also cause encephalitis, a severe and usually fatal infection of the central nervous system in immunocompromised individuals [4,6].

*Acanthamoeba* species have initially been divided into three morphological groups (I, II, and III), with clinical isolates commonly belonging to group II [7]. Subsequently, *Acanthamoeba* spp. have been classified into 23 genotypes (T1-T23) based on their 18S rRNA whole gene sequences [8,9,10,11]. Genotype T4 with its currently seven main 18S subtypes (T4A–T4G) is the most prevalent genotype [12]. Despite being the most common genotype isolated from the environment, T4 is still over-represented in human disease [13]. However, genotypes T3, T5, T6, T11, and T15 have also repeatedly been isolated from patients [4,9,14].

The diagnosis of *Acanthamoeba* infections by microscopic examination of clinical samples is challenging and requires skilled laboratory personnel. In addition, prolonged incubation of cultures may be necessary, particularly when patients have been treated already [15]. However, *Acanthamoeba* diagnosis has been facilitated and improved by the establishment of several PCR assays targeting various regions of the nuclear small subunit 18S rRNA gene (Rns) [5,16,17,18,19,20]. Our present study aimed to develop a rapid and reliable qPCR assay to precisely detect and synchronously quantify the predominant *Acanthamoeba* genotype T4. For evaluation, the new assay was compared to two published assays and one commercial kit used in *Acanthamoeba* diagnosis.

## 2. Materials and Methods

***Acanthamoeba* strains**. *Acanthamoeba* strains T4 (WIR17, 3ST), T5 (72-2 and AFR6), T6 (11DS), T10 (70171), T11 (Zoo9), T12 (BUD9), and 22 positive corneal samples in culture from our culture collection were used to develop the T4 genotyping method (Table S1). The strains were maintained on non-nutrient *E. coli*-coated agar plates [4]. In addition, two T4 strains (STR16 and WIR17) and one T5 strain (72-2) maintained in culture flasks as described previously [4] were counted and used as reference strains to evaluate and compare the study methods. The genotypes of the strains used were re-confirmed by sequencing the fragment obtained with the standard JDP1–JDP2 primers [17] (Figure S1).

**Clinical samples and DNA extraction.** The methods were evaluated using DNA from 185 clinical corneal scrapings obtained between 2015 and 2021. The standard diagnostic tests of the clinical samples at our institution included microscopic analysis of the samples, culture on *E. coli*-coated agar plates evaluated daily for seven days, and JDP standard PCR [17]. All samples were anonymized, and DNA was extracted using the tissue protocol of QIAamp DNA mini kit (Qiagen, Hilden, Germany).

**Primers, Probes, and qPCR procedure.** qPCR was performed with all samples using the newly designed *Acanthamoeba* genotype T4 primers and probe, as also those from Qvarnstrom and colleagues and Karsenti and colleagues [18,20]. For the new T4 assay, a fragment of the 18S small subunit ribosomal RNA (SSU rRNA) gene was targeted to selectively detect genotype T4. The primers AcT4F and AcT4R and the hydrolysis probe AcT4P were designed using the Primer3 online tool (http://bioinfo.ut.ee/primer3-0.4.0/, accessed on 15 September 2020) and checked for specificity using the Basic Local Alignment Search Tool (BLAST). Additionally, published primers and probes established by Qvarnstrom et al. [18] (AcantF900, AcantR1100, AcantP1000), and Karsenti et al. [20] (AcanthF3, AcanthR2, AcanthP2) also targeting the 18S rRNA gene were used. The sequences of the primers and probes used in this study are shown in Table S2. The T4 primers and probe were synthesized by Eurofins MWG Synthesis GmbH (Ebersberg, Germany). Primers and probes for the other assays were ordered from Microsynth GmbH (Vienna, Austria). Twenty-four additional clinical samples were used to compare the assays with the commercial kit ParoReal Kit *Acanthamoeba* (Ingenetix GmbH, Vienna, Austria).

All three qPCR methods were run in total volumes of 20 µL containing primers (0.5 µM), probes (0.1 µM), 1× qPCR master mix (BioRad, Hercules, CA, USA), and 4 μL DNA extract. PCRs were performed on a CFX Connect real-time PCR detection system (BioRad Laboratories, Inc., Singapore) with an initial denaturation step at 95 °C for 3 min, followed by 40 cycles of 95 °C for 10 s and 60 °C for 50 s. The same proportion of DNA in the PCR mix was used with the ParoReal kit to make all results comparable.

**Assays specificity and sensitivity.** The specificity of the “Qvarnstrom” (Qvar) and “Karsenti” (Kars) assays were already described by the authors [18,20]. The specificity of our T4 primers and probe was confirmed by BLAST search. The T4 assay specificity was also evaluated by using the 22 positive corneal culture samples (Table S1).

The efficiency of the PCR amplification was assessed with the mean of three calibration curves with serial dilutions of amoebae from 10^5^ to 1 cells/µL of each strain (STR16, WIR17, and 72-2). The PCR efficiency was calculated according to a previously described study [21]. The limit of detection (LOD) of all three *Acanthamoeba* strains was evaluated as the measured concentration producing at least 95% positive replicates [21,22]. The LOD was determined by amplifying seven concentrations (200, 100, 50, 20, 10, 5, 1 cells/µL) of each *Acanthamoeba* strain four times six (24 replicates). The LOD is the lowest number of amoebae required to be detected by the PCR and in our study one cell means one amoeba. The total proportion of positive tests was subjected to probit regression analysis using R version 4.0.0 (24 April 2020) via RStudio version 1.3.959 to obtain the LOD with confidence interval (CI). For pairwise agreement analysis, the Cohen-kappa tests were performed using the Visualizing Categorical Data (vcd) package in R. The quantification cycle (Cq) distribution plots were produced using the ggplot 2 package in R, and the UpSet graph was produced using the package ‘UpSetR’ in R.

## 3. Results

The newly designed T4 forward and reverse primers (Table 1) were selected to specifically amplify all *Acanthamoeba* T4 sub-types (T4A–T4G). No amplification was observed for *Acanthamoeba* genotypes T3, T5, T6, T10, T11, and T12 from our culture collection. The primers were also submitted to BLAST search to confirm their specificity. The sensitivities of the T4 assay and the Qvar and Kars assays were evaluated by individually amplifying tenfold serial dilutions (10^5^–1 cell/µL) of parasite count of the same strains at the same concentrations. The efficiencies for the *Acanthamoeba* strains (STR16-T4, WIR17–T4, and 72-2-T5) ranged from 92.01 to 97.59%, (slope −3.3810 to −3.5703, and R^2^ of 0.9768 to 0.9951) for the tenfold serial dilutions (Table 1). LODs were determined with further dilutions between 200 and 1 parasites/µL. The calculated LOD range at 95% confidence using probit analysis was 3.63 (2.12–62.04) with T4 primers to 33.27 (19.34–78.65) with the Kars primers (Table 1).

**Figure 1:**
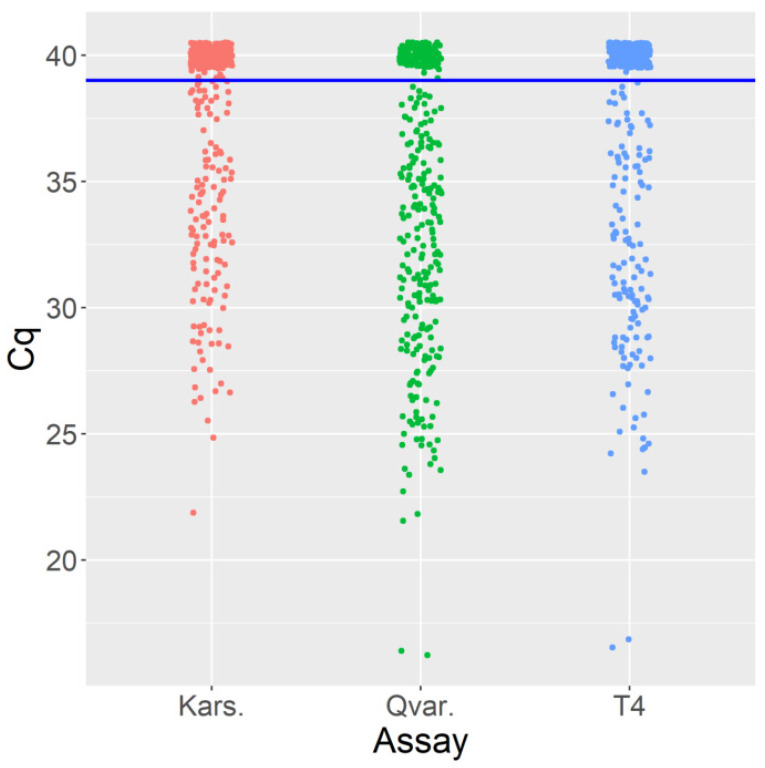
Cq values distribution plot of 185 clinical corneal samples with Kars assay (red), Qvar assay (green), and T4 assay (blue). The individual Cq values of each assay are plotted to show an overall Cq pattern and the range of the Cq values. The Cq of negative samples were arbitrarily set at 40 for visualization purpose.

The validation of the discrimination power of the assays was tested using 185 archived DNA samples from clinical material of suspected AK patients obtained between 2015 and 2021. Figure 1 presents the samples’ quantitative cycle (Cq) values distribution with all three assays. Thus, the Qvar assay shows the lowest Cq values, proving its higher sensitivity than the other assays. The Cq distribution of the T4 assay was similar to that of the Qvar assay but with less density (Figure 1).

**Figure 2:**
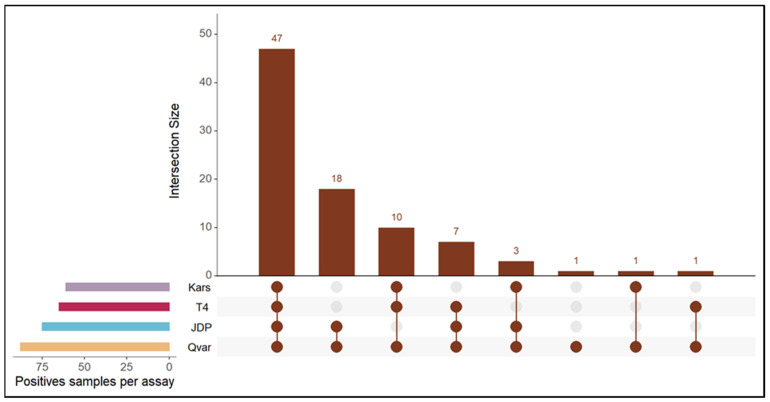
Upset graph of the assays presenting the size of the intersections (cardinality) of the positive samples with JDP (turquoise), Qvar (orange), Kars (purple), and T4 (red) assays.

Interestingly, the proportion of positive samples (47.57%) using the Qvar method was similar to that of the standard JDP primers (40.54%) with an excellent Cohen’s kappa agreement (0.82, *p* < 0.05) beyond chance, according to Fleiss et al. [23] classification (Figure S3). The Kars assay was less sensitive compared to Qvar with only 32.97% positive rate (Figure 2) with a good (0.72, *p* < 0.05) and a fair (0.57, *p* < 0.05) agreement beyond chance with Qvar and JDP assays, respectively. In the new T4 assay 35.14% of the tested samples were positive; however, of course, this assay only detects T4. Comparing the results of the different assays using the 185 archived DNA samples, 88 were positive in the Qvar assay. Half of the positive samples (53.41%) were detected by all four assays. Three samples that tested positive with JDP, Qvar, and Kars assays, were negative in the new T4 assay (Figure 2). Altogether, 23 samples that were positive in Qvar and/or JDP and/or Kars were negative in the T4 assay (Figure 2). These samples, however, may be non-T4 genotypes, which could be confirmed for those that gave amplicons suitable for DNA sequencing in the JDP assay.

The JDP PCR products of three non-T4 samples were sequenced and confirmed to be T11 (1 sample) and T3 (2 samples).

**Figure 3:**
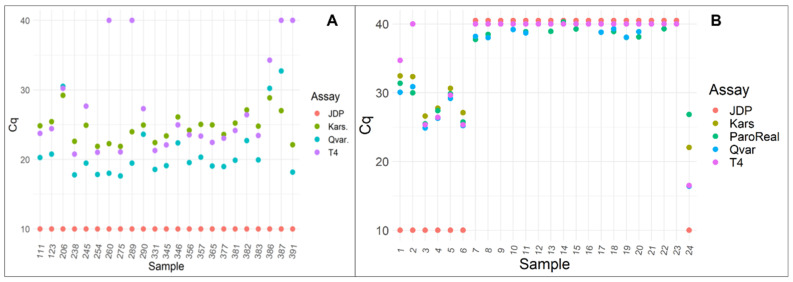
Cq values of positive corneal culture samples (**A**), and from direct clinical corneal samples (**B**). With a Cq cut off of 37 in (**B**), all methods (JDP, Qvar, Kars, and T4) present the same results, except for one sample that was not detected by the T4 assay and was found to be a T3 by sequencing. Dots represent the JDP positive samples with Cq values set at 10, while the negative samples are illustrated with dots at a Cq value of 40.

For a more detailed evaluation of the specificity of our T4 primers, we tested the primers with 22 positive corneal culture samples. The results revealed four non-T4 samples and matched with the sequencing data of these 22 culture samples (Table S1). The Cq values of the positive corneal culture samples are presented in Figure 3A. Figure 3B shows the Cq values of selected corneal samples used to compare the Qvar and Kars assays with the ParoReal *Acanthamoeba* kit. An overall high Cohen’s kappa agreement beyond chance was found between the ParoReal kit and Qvar (0.81) and Kars (0.81), respectively.

## 4. Discussion

The gold standard for *Acanthamoeba* diagnosis is still culture on *E. coli*-coated non-nutrient agar plates. However, in the past years, several standard PCR or real-time PCR protocols have become available. In most parts of the world, the vast majority of *Acanthamoeba* infections, including *Acanthamoeba* keratitis (AK) and Granulomatous Amebic Encephalitis (GAE), have been caused by genotype T4 [4]. Therefore, we aimed to develop a new real-time PCR assay to precisely and rapidly detect and quantify this genotype in clinical samples. This study established a real-time PCR to diagnose *Acanthamoeba* and determine the genotype T4 simultaneously. The challenge for developing this method was to design a primer set targeting all T4 subtypes (T4A–T4G) but being specific for genotype T4 only.

Our data demonstrate that the new T4 primers detected all samples that were known to be T4. The specificity of this new assay is based on the forward primer F1 binding specifically to all T4 sub-types (T4A–T4G). The uniqueness of the novel T4 assay is that the method is targeting directly and only the T4 genotype, thus allowing T4 identification without the need for sequencing. Recently, Holmgaard and collaborators [24] established a Next Generation Sequencing (NGS) 16S-18S assay, which provide information on *Acanthamoeba* genotypes by analysis of the sequencing data. This NGS assay provides more data, but is more costly, and requires more skilled personnel to perform bioinformatics analyses.

In the current study, we also prospectively compared the performance of Qvar and Kars assays on clinical samples to select one assay to be run in duplex with our T4 assay. A good agreement (0.72) beyond chance was obtained between the two assays according to Fleiss et al. [23]. The discrepancy of 27 positive samples between the two assays could be due to a lower sensitivity of Kars assay or to the c-terminal position of Kars target fragment (127 bp). Both primer sets are well located on conserved regions, with the Qvar fragment (177 bp) having fifty base pairs more and being centrally well located in the 18S rDNA sequence. This could plausibly explain the high sensitivity of the Qvar assay. The good performance of the Qvar assay was also confirmed by other authors [20,25]. Comparison of the Qvar and Kars assays with the ParoReal *Acanthamoeba* kit using cultured samples, demonstrated high agreement beyond chance between the assays, further proving the suitability of the Qvar assay for low positive samples.

Running the Qvar assay in duplex with the novel T4 assay would allow rapid *Acanthamoeba* diagnosis in one run, detecting all genotypes and synchronously identifying and quantifying T4, which accounts for the large majority of human infections. This would provide rapid and detailed information on the causative agent and allow for a timely case management.

A limitation of our study was that only seven genotypes (T3, T4, T5, T6, T10, T11, T12) of 23 described genotypes were included in the test runs. However, in silico analysis aligning all 23 genotypes confirmed that only T14 might require further validation (Figure S2).

## 5. Conclusions

We successfully developed and validated a novel qPCR assay targeting *Acanthamoeba* genotype T4, which can be run in duplex with the Qvarnstrom et al. [18] assay to detect and quantify Acanthamoeba genotype T4 and all other genotypes in clinical samples rapidly, efficiently, and simultaneously. The developed method could also be used for epidemiological surveys and contribute significantly to improving *Acanthamoeba* infection control. However, the novel assay still requires further inter-genotype and inter-laboratory validation before implementation into routine diagnosis.

## Figures and Tables

**Table 1:** Assay Efficiency, and limits of detection (LOD).

qPCR Method	Acanthamoeba Genotype	Efficiency (%)	Slope	R2	LOD with CI (Cell ^1^/µL)
Kars	STR16 (T4)	94.25	−3.4680	0.9768	5.28 (3.20–22.89)
	WIR17 (T4)	94.66	−3.4568	0.9821	33.27 (19.34–78.65)
	72-2 (T5)	90.58	−3.5703	0.9952	6.10 (4.31–10.63)
Qvar	STR16 (T4)	93.89	−3.4775	0.9951	3.99 (1.74–22.88)
	WIR17 (T4)	97.59	−3.3810	0.9907	10.26 (5.80–32.99)
	72-2 (T5)	94.03	−3.4738	0.9887	4.86 (3.42–8.49)
T4	STR16 (T4)	92.01	−3.5295	0.9901	3.63 (2.12–62.04)
	WIR17 (T4)	92.27	−3.5223	0.9906	15.89 (9.20–41.89)

^1^ Cell = Amoebae.

## Data Availability

The data presented in this study are available in supplementary material.

## Supplementary Materials

The following supporting information can be downloaded at: https://www.mdpi.com/article/10.3390/microorganisms10071307/s1, Figure S1: Sequencing Results *Acanthamoeba* strains used for primers validation; Figure S2: Alignment sequences of the 23 genotypes of *Acanthamoeba* with the position of the T4 primers shown in red; Figure S3: PCR results of the 185 clinical corneal samples with JDP, Kars, Qvar, and T4 assays; Table S1: List of sequenced samples with their genotype and strain; Table S2: List of primers and probes.
