# Supplementary material for: Real-Time PCR for the Diagnosis of Acanthamoeba Genotype T4
Source: Microorganisms. 2022 Jun 28;10(7):1307. doi: 10.3390/microorganisms10071307 (PMC9325200; doi:10.3390/microorganisms10071307)
Supplement: Supplementary file 1 [file microorganisms-10-01307-s001.zip › microorganisms-1761848-supplementary.pdf]

Supplementary Files

Real-time PCR for the diagnosis of *Acanthamoeba* genotype T4

Aline Lamien-Meda [1]*, Martina Köhsler [1] and Julia Walochnik [1]

Table S1. List of sequenced samples with their genotype and strain.

Sample Code	Genotype	Strain
97	T4	3ST
127	T11	ZOO9
89	T6	11DS
103	T5	72-2
306	T5	AFR6
110	T12	BUD9
231	T10	70171
331	T4	STR16
111	T4	DU08
123	T4	SPA08
206	T4	Pes09
238	T4	Dih12
245	T4	Lar12
254	T4	Ume13
260	T6	Sck13
275	T4	Jäl14
289	T4	Str15
290	T4	Obr15
331	T4	STR16
345	T4	Wir17
346	T4	N17
356	T4	Tel18
357	T4	Rur18
365	T4	Stgl19
377	T4	Fea19
381	T4	XIN20
382	T4	MAI20
383	T4	Sin20
386	T4	Schmidt21
387	T6	Häg21
391	T3	Dab 21

Table S2. List of primers and probes.

Assay	Primers and Probe	Fragment size (bp)
JDP1-DP2	JDP1: 5'-GGCCCAGATCGTTTACCGTGAA-3'<br>JDP2: 5'-TCTCACAAGCTGCTAGGGAGTCA-3'	423-551
Qvarnstrom	AcantF900: 5′-CCC AGA TCG TTT ACC GTG AA-3′<br>AcantR1100: 5-AAT ATT AAT GCC CCC AAC TAT CC-3′<br>AcantP1000: 5-FAM-CTGCCACCGAATACATTAGCATGG- [BHQ1]-3′	177
Karsenti	AcanthF3: 5′-GGTCCGGGTAATCTTTGCAAA-3′<br>AcanthR2: 5'- GTACAAAGGGCAGGGACGTAATCAAC-3′<br>AcanthP2: 5'-FAM- TAAGCGCGAGTCATC-1 [MGB]- 3′	127
T4 genotype This study	AcT4F1: 5'- GCTTCGCGGCGACGTCATC-3'<br>AcT4R1: 5'- CTCAAACTTCCGCTGGCTAGG-3'<br>AcT4P: 5'-HEX-GTTCGGCGTCCGTGCTTCTTAGAG-1 [BHQ1]	112
Ingenetix	Commercial kit	

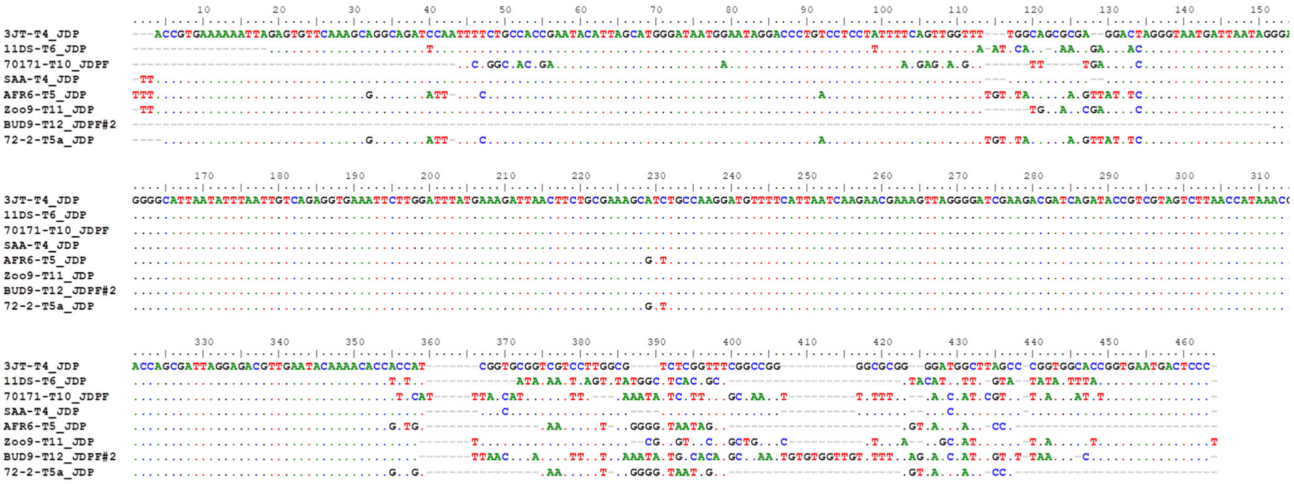

Figure S1. Sequencing Results Acanthamoeba strains used for primers validation: 3JT(T4), 11DS (T6), 70171(T10), SAA (T4), AFR6 (T5), Zoo9 (T11), BUD9 (T12), 72-2 (T5)

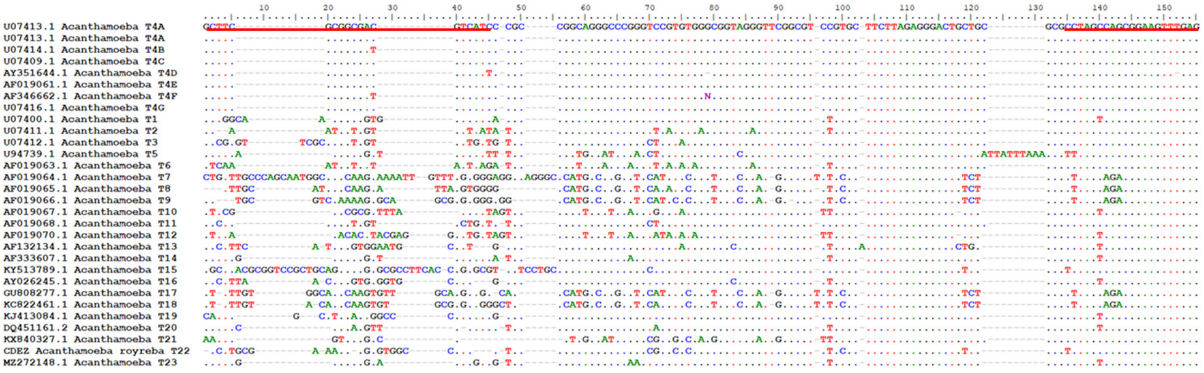

Figure S2. Alignment sequences of the 23 genotypes of *Acanthamoeba* with the position of the T4 primers shown in red.

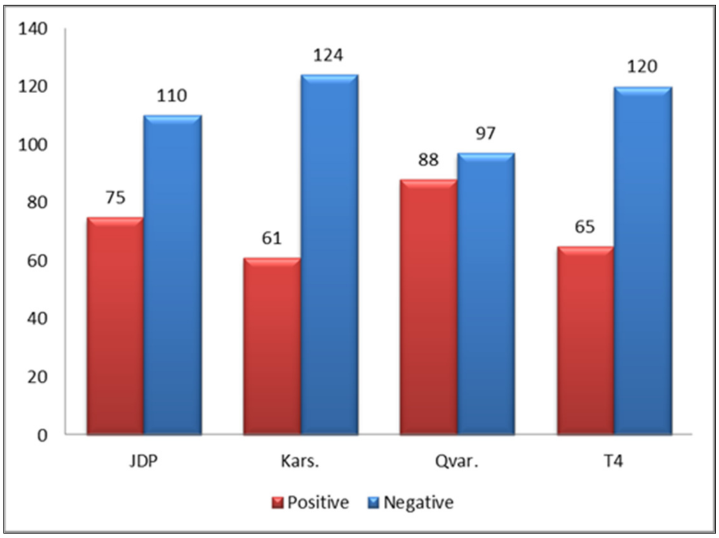

Figure S3. PCR results of the 185 clinical corneal samples with JDP, Kars., Qvar., and T4 assays. The numbers of positive samples for each assay are shown in red, and that of negative samples in blue. The "Qvar." assay was the most sensitive with 47.6% positive samples, followed by JDP assay (40.54%), T4 assay (35.14%), and Kars. assay (32.97%).

Sequencing data:

>3JT-T4_JDP

Accgtgaaaaaattagagtgttcaaagcaggcagatccaattttctgccaccgaatacattagcatgggataatggaataggaccctgtcctcct
attttcagttggttttggcagcgcgaggactagggtaatgattaatagggatagttgggggcattaatatttaattgtcagaggtgaaattcttgga
tttatgaaagattaacttctgcgaaagcatctgccaaggatgttttcattaatcaagaacgaaagttaggggatcgaagacgatcagataccgtc
gtagtcttaaccataaacgatgccgaccagcgattaggagacgttgaatacaaaacaccaccatcggtgcggtcgtccttggcgtctcggtttcgg
ccggggcgcggggatggcttagcccggtggcaccggtgaatgactccc

>11DS-T6_JDP

gagtgttcaaagcaggcagattcaattttctgccaccgaatacattagcatgggataatggaataggaccctgtcctcctttttcagttggttaatt
cacacaagagaggaacagggtaatgattaatagggatagttgggggcattaatatttaattgtcagaggtgaaattcttggatttatgaaagatt
aacttctgcgaaagcatctgccaaggatgttttcattaatcaagaacgaaagttaggggatcgaagacgatcagataccgtcgtagtcttaacca
taaacgatgccgaccagcgattaggagacgttgaatacaaaacacctctatatataattcagtgtatggcttcacggctgtacattgtttgtatata
gtttacggtgaatgactccc

>72-2-T5_JDPR2

gagtgttcaaagcaggcagattcaattttctgccaccgaatacattagcatgggataatggaataggaccctgtcctcctttttcagttggttaatt
cacacaagagaggaacagggtaatgattaatagggatagttgggggcattaatatttaattgtcagaggtgaaattcttggatttatgaaagatt
aacttctgcgaaagcatctgccaaggatgttttcattaatcaagaacgaaagttaggggatcgaagacgatcagataccgtcgtagtcttaacca

taaacgatgccgaccagcgattaggagacgttgaatacaaaacacctctatatataattcagtgtatggcttcacggctgtacattgtttgtatata
gtttacggtgaatgactccc

>70171-T10_JDPF

ttctggccaccgaaatacattagcatgggataatgaaataggaccctgtcctcctatttacgagtaggtttttgaggaccagggtaatgattaata
gggatagtcgggggcattaatatttaattgtcagaggtgaaattcttggatttatgaaagattaacttctgcgaaagcatctgccaaggatgttttc
attaatcaagaacgaaagttaggggatcgaagacgatcagataccgtcgtagtcttaaccataaacgatgccgaccagcgattaggagacgttg
aatacaaaacaccatccatttagcatggtcgttttcaaatattcctttttgcgaaggttgtttgggaacgattcgtcctgatggatctggtgaatgac
tccc

>SAA-T4_JDP

ttaccgtgaaaaaattagagtgttcaaagcaggcagatccaattttctgccaccgaatacattagcatgggataatggaataggaccctgtcctcc
tattttcagttggttttggcagcgcgaggactagggtaatgattaatagggatagttgggggcattaatatttaattgtcagaggtgaaattcttgg
atttatgaaagattaacttctgcgaaagcatctgccaaggatgttttcattaatcaagaacgaaagttaggggatcgaagacgatcagataccgt
cgtagtcttaaccataaacgatgccgaccagcgattaggagacgttgaatacaaaacaccaccatcggcgcggtcgtccttggcgtctcggtttc
ggccggggcgcggggacggcttagcccggtggcaccggtgaatgactccc

>AFR6-T5_JDP

tttaccgtgaaaaaattagagtgttcaaagcgggcagatatttttcctgccaccgaatacattagcatgggataatggaataggaccctgacctcc
tattttcagttggttttgtttacagcgaggttatatcagggtaatgattaatagggatagttgggggcattaatatttaattgtcagaggtgaaattct
tggatttatgaaagattaacttctgcgaaagcgtttgccaaggatgttttcattaatcaagaacgaaagttaggggatcgaagacgatcagatac
cgtcgtagtcttaaccataaacgatgccgaccagcgattaggagacgttgaatacaaaacaccgctgttaatcctttcgggggttaatagttggtg
aatgactccc

>Zoo9-T11_JDP

Ttaccgtgaaaaaattagagtgttcaaagcaggcagatccaattttctgccaccgaatacattagcatgggataatggaataggaccctgtcctc
ctattttcagttggttttggcacgcgaggaccagggtaatgattaatagggatagttgggggcattaatatttaattgtcagaggtgaaattcttgg
atttatgaaagattaacttctgcgaaagcatctgccaaggatgttttcattaatcaagaacgaaagttaggggatcgaagacgatcagataccgt
cgtagtcttaaccataaacgatgccgaccagcgattaggagacgttgaatacaaaacaccaccatcggtgcggtcgtccttggcgcgtcgtggct
tgctgcggcgtgcgagggcgatttagcctgatggcatcggtgaatgactccct

>BUD9-T12_JDPF#2

Ggatagttgggggcattaatatttaattgtcagaggtgaaattcttggatttatgaaagattaacttctgcgaaagcatctgccaaggatgttttca
ttaatcaagaacgaaagttaggggatcgaagacgatcagataccgtcgtagtcttaaccataaacgatgccgaccagcgattaggagacgttga
atacaaaacaccaccattaacgcgatcgtttttttgcaaatattgccacatgcgcaagtgtgtggttgtgtttggaggaacgatttgtcttaatggcc
cggtgaatgactccc

>Hni_T11

AATATTGGATCTGCCTGCTTTGAACACTCAGATTTATGAAAGATTAACTTCTGCGAAAGCATCTGCCAAGGATG
TTTTCATTAATCAAGAACGAAAGTTAGGGGATCGAAGACGATCAGATACCGTCGTAGTCTTAACCATAAACGA
TGCCGACCAGCGATTAGGAGACGTTGAATACAAAACACCACCATCGGTGCGGTCGTCCTTGGCGCGTCGCGGC

TTGCCGCGGCGTGCGAGGGCGATTTAGCCTGATGGCATCGGTGAATGACTCCCAGCACTTTGTGAGAAABGGCCCAAA

>WE _T4

GCCTGCTTTGATCACTCAGATTTATGAAAGATTAACTTCTGCGAAAGCATCTGCCAAGGATGTTTTCATTAATCAAGAACGAAAGTTAGGGGATCGAAGACGATCAGATACCGTCGTAGTCTTAACCATAAACGATGCCGACCAGCGATTAGGAGACGTTGAATACAAAACACCACCATCGGTGCGGTCGTCCTTGGCGTCTCGGTTTCGGCCGGGGTGCGGGGACGGCTTAGCCCGGTGGCACCGGTGAATGACT

>Contig\F2-E2_289_T4

Tagcagcttgtgagatttttggcccagatcgtttaccgtgaaaaaattagagtgttcaaagcaggcagatccaattttctgccaccgaatacattagcatgggataatggaataggaccctgtcctcctattttcagttggtttgccgcgaggaccagggtaatgattaatagggatagttgggggcattaatatttaattgtcagaggtgaaattcttggatttatgaaagattaacttctgcgaaagcatctgccaaggatgttttcattaatcaagaacgaaagttaggggatcgaagacgatcagataccgtcgtagtcttaaccataaacgatgccgaccagcgattaggagacgttgaatacaaaacaccaccatgcgcattgcggtcgtctttggtgtcgctcacaaggcggcaccggggcggcttagctcgcatggcaccggtgaatgactccctagcagctg
